# Lipid changes due to fenofibrate treatment are not associated with changes in DNA methylation patterns in the GOLDN study

**DOI:** 10.3389/fgene.2015.00304

**Published:** 2015-09-29

**Authors:** Mithun Das, M. Ryan Irvin, Jin Sha, Stella Aslibekyan, Bertha Hidalgo, Rodney T. Perry, Degui Zhi, Hemant K. Tiwari, Devin Absher, Jose M. Ordovas, Donna K. Arnett

**Affiliations:** ^1^Department of Epidemiology, School of Public Health, University of Alabama at BirminghamBirmingham, AL, USA; ^2^Department of Biostatistics, Section on Statistical Genetics, School of Public Health, University of Alabama at BirminghamBirmingham, AL, USA; ^3^Absher Laboratory, HudsonAlpha Institute of BiotechnologyHuntsville, AL, USA; ^4^Nutrition and Genomics Laboratory, Jean Mayer USDA Human Nutrition Research Center on Aging, Tufts UniversityBoston, MA, USA

**Keywords:** fenofibrate, lipid lowering drug, epigenetic changes, DNA methylation, dyslipidemia, cardiovascular disease

## Abstract

Fenofibrate lowers triglycerides (TG) and raises high density lipoprotein cholesterol (HDLc) in dyslipidemic individuals. Several studies have shown genetic variability in lipid responses to fenofibrate treatment. It is, however, not known whether epigenetic patterns are also correlated with the changes in lipids due to fenofibrate treatment. The present study was therefore undertaken to examine the changes in DNA methylation among the participants of Genetics of Lipid Lowering Drugs and Diet Network (GOLDN) study. A total of 443 individuals were studied for epigenome-wide changes in DNA methylation, assessed using the Illumina Infinium HumanMethylation450 array, before and after a 3-week daily treatment with 160 mg of fenofibrate. The association between the change in DNA methylation and changes in TG, HDLc, and low-density lipoprotein cholesterol (LDLc) were assessed using linear mixed models adjusted for age, sex, baseline lipids, and study center as fixed effects and family as a random effect. Changes in DNA methylation were not significantly associated with changes in TG, HDLc, or LDLc after 3 weeks of fenofibrate for any CpG. CpG changes in genes known to be involved in fenofibrate response, e.g., *PPAR*-α, *APOA1, LPL, APOA5, APOC3, CETP*, and *APOB*, also did not show evidence of association. In conclusion, changes in lipids in response to 3-week treatment with fenofibrate were not associated with changes in DNA methylation. Studies of longer duration may be required to detect treatment-induced changes in methylation.

## Introduction

Fenofibrate is a peroxisome proliferator-activated receptor-α (PPAR-α) agonist which improves lipid profiles, particularly by reducing triglycerides (TG) and increasing high density lipoprotein cholesterol (HDLc) (Schoonjans et al., 1996; Staels et al., 1998). The effect of fenofibrate on the metabolism of TG-rich lipoproteins is due to PPAR-α-dependent stimulation of the lipoprotein lipase (*LPL*), apolipoprotein A-I (*APOA1*), and apolipoprotein A-V (*APOA5*) genes, and inhibition of the apolipoprotein C-III gene (*APOC3*) to decrease TG. The increase in plasma HDLc depends on overexpression of *APOA1* as well as down regulation of the apolipoprotein B (*APOB*) (Auwerx et al., 1996; Staels et al., 1997; Fruchart and Duriez, 2006; Aslibekyan et al., 2015) and cholesteryl ester transfer protein, plasma (*CETP*) genes (van der Hoogt et al., 2007).

The effect of pharmacologic treatment on lipids shows considerable inter-individual differences, making studies of genetic predictors of fenofibrate response worthwhile. In previous GOLDN (Genetics of Lipid Lowering Drug and Diet Network) publications, common genetic polymorphisms were found to be associated with variation in fenofibrate response. Findings included genes encoding the scavenger receptor class B, member 1 (*SCARB1*), a key component in the reverse cholesterol transport (Liu et al., 2008); *APOA5*, an important player in lipid metabolism and homeostasis (Lai et al., 2007); ATP-binding cassette, sub-family A (ABC1), member 1 (*ABCA1*), involved in cellular lipid efflux and HDL metabolism (Tsai et al., 2010); *APOB*, essential for transfer of TG and cholesteryl esters during lipoprotein metabolism (Wojczynski et al., 2010); and peroxisome proliferator-activated receptor alpha (*PPARA*), involved in the pharmacodynamic pathway (Frazier-Wood et al., 2013).

Though there have been multiple genetic studies, there are biochemical reasons to suspect that DNA methylation may be an important factor in TG response to fenofibrate. *PPARA* is known to regulate homocysteine production (Foucher et al., 2010). In fact, one of the side effects of fenofibrate treatment (which targets PPARα) is elevated homocysteine (Foucher et al., 2010). Homocysteine is a component of the methionine biosynthetic pathway, which is responsible for the production of S-adenosylmethionine, the source of methyl groups for DNA methylation. Furthermore, folate, which has been shown to influence DNA methylation levels, is a coenzyme in this pathway, and dietary folate is effective in abrogating the hyperhomocysteinemia induced by fenofibrate (Foucher et al., 2010). We propose that lipid metabolic pathways and DNA methylation pathways are linked and perturbations in one may directly influence the other. For these reasons, we hypothesized that fenofibrate could induce epigenetic alterations.

Our research is also motivated by other studies which have suggested interventions targeting improvements in cardio-metabolic health are associated with changes in DNA methylation (Crujeiras et al., 2013; Rönn et al., 2013; Deiuliis et al., 2014; Jacobsen et al., 2014; Martin-Núñez et al., 2014; Milenkovic et al., 2014; Su et al., 2014; Benton et al., 2015). Many of these studies have been candidate gene studies and/or global DNA methylation studies, yet they support continued investigation of DNA methylation changes and metabolic response to diet and drug interventions. For example in a mediterranean diet intervention study, global DNA methylation as well as methylation in the promoter region of the stearoyl-CoA desaturase (delta-9-desaturase) (SCD) gene was similar at baseline but different between the control and intervention groups at study conclusion. The authors concluded DNA methylation is associated with weight change and diet adherence.

To address whether a 3-week fenofibrate intervention is associated with changes in DNA methylation during that time period, we conducted a genome-wide study of DNA methylation changes using data collected before and after fenofibrate on lipids and from the Illumina Infinium HumanMethylation450 Beadchip set within the Genetics of Lipid Lowering Drugs and diet network (GOLDN) study. We also conducted secondary analysis focused on candidate genes belonging to the fenofibrate response pathway, specifically *PPARA, APOA1, LPL, APOA5, APOC3, CETP*, and *APOA*.

## Methods

### Study population

The GOLDN study comprises families of European descent recruited from two field centers in Minneapolis, MN and Salt Lake City, UT. As part of the NHLBI Family Heart Study, GOLDN was designed to identify genetic determinants of lipid response to two interventions: (1) a high-fat meal challenge and (2) treatment with fenofibrate (160 mg) for 3 weeks. Families with at least two siblings were recruited (*n* = 1327 individuals) and were required to withhold lipid-lowering medications for at least 4 weeks prior to the initial visit. A total of 1053 individuals met all eligibility requirements. Validated questionnaires were used to collect demographic, lifestyle (smoking and alcohol), and dietary data [Diet History Questionnaire (DHQ)] as described in previous publications (Smith et al., 2009; Wood et al., 2011). Written consent was obtained from each participant during the screening visit. This protocol was approved by Institutional Review Boards of University of Minnesota, University of Utah, Tufts University/New England Medical Center, and the University of Alabama at Birmingham. Details of the GOLDN study are available in previous publications (Feitosa et al., 2011; Aslibekyan et al., 2012; Frazier-Wood et al., 2014; Hidalgo et al., 2014; Irvin et al., 2014). Baseline and post-intervention epigenetic data were available for 443 (215 men and 228 women) participants from 139 families.

### Lipid profiles

Lipids were measured both before and after 3 weeks of fenofibrate intervention. TG levels were measured by glycerol blanked enzymatic method (Trig/GB, Roche Diagnostics Corporation, Indianapolis, IN). Low-density lipoprotein cholesterol (LDLc) was measured by a homogenous direct method (LDL Direct Liquid Select Cholesterol Reagent, Equal Diagnostics, Exton, PA). HDLc was calculated after precipitation of non-HDLc with magnesium/dextran.

### DNA methylation

For methylation assays, CD4+ T cells were harvested from stored buffy coats using antigen-specific magnetic beads (Invitrogen, Carlsbad, CA). DNA was extracted using DNeasy kits (Qiagen, Venlo, Netherlands). We used the Illumina Infinium HumanMethylation450 Beadchip (Illumina Inc., San Diego, CA) to quantify genome-wide DNA methylation described in detail in a previous publication (Absher et al., 2013). For each assay, 500 ng of DNA was treated with sodium bisulfite (EZ DNA, Zymo Research, Irvine, CA) before standard Illumina amplification, hybridization, and imaging steps. We used Illumina Genome Studio to estimate β scores (the proportion of total signal from methylation-specific probe or color channel) and detection *P*-values [probability that the total intensity for a given probe falls within the background signal intensity (Illumina GenomeStudio)]. In the quality control (QC) stage, β-scores with an associated detection *P*>0.01 were removed and samples with >1.5% missing data points were eliminated from further analysis. Moreover, any CpG probes for which >10% of samples failed to yield adequate intensity were removed. These methods are described extensively in Absher et al. (2013). Due to observed batch effects the filtered β scores were normalized using the ComBat package for R software (Chen et al., 2011). We performed the normalization on random subsets of 10,000 CpGs per run, in which each array of 12 samples was used as batch. To correct for differing probe chemistry on Illumina Infinium HumanMethylation450 Beadchip, we separately normalized probes form the Infinuim I and II as mentioned in our previous publications (Hidalgo et al., 2014; Irvin et al., 2014) with the detailed description in Absher et al. (2013). We further eliminated any CpGs in which the probe sequence mapped either to a location that did not match the annotation file or to >1 locus. We identified such markers by realigning all probes (with unconverted Cs) to the human reference genome. After QC procedures, there were methylation data from 461,281 CpGs. Principal components (PCs) based on the β-scores of all autosomal CpGs passing QC were generated using the *prcomp* function in R (v2.12.1). The methylation assay for the 443 samples collected after fenofibrate intervention was performed in a similar way.

### Statistical methods

Differences in lipid profiles pre- and post-fenofibrate intervention were analyzed using paired *t*-tests. To remove the confounding due to T-cell impurity and batch effects in DNA methylation profiles, we obtained residuals of methylation by first modeling the four PCs (to account for T-cell purity) derived from whole-genome methylation as fixed effects and batch as random effect in linear mixed models for both pre- and post-intervention measurements. Previous work in GOLDN has demonstrated the usefulness of adjusting for four PCs to account for T-cell purity (Irvin et al., 2014). The change in methylation after the 3 week intervention was assessed as the change in methylation residual (described above) which was calculated as the difference between the residuals computed from methylation data obtained before and after fenofibrate. Linear mixed models were used to investigate association between change in methylation residual and changes in TG, HDLc, and LDLc. The primary models adjusted for age, sex, and center as fixed effects and family as a random effect using the *lmekin* function of the kinship package in R (Atkinson and Therneau, 2003). In sensitivity analysis, we additionally adjusted for lifestyle factors that may modify DNA methylation including smoking (current smoking yes or no), alcohol consumption (in grams per day), and fat intake (in grams per day). We used the Bonferroni correction to adjust for multiple comparisons, with the genome-wide significance level of 0.05/(3^*^461281) = 3.6 × 10^−8^. We constructed Manhattan plots to visualize the results. To evaluate deviations from the expected test statistic distributions we constructed quantile-quantile (Q-Q) plots. Finally, for the seven known candidate genes associated with fenofibrate response, we identified and tested 104 CpGs in secondary analysis. The adjusted significance level was determined to be 0.05/(3 × 104) = 1.6 × 10^−4^. To estimate the effect size of the methylation change variable on lipid response for our top findings we selected the maximum number of unrelated individuals from our data (*N* = 158) and report the difference in the *R*^2^ estimate from a linear model with and without the CpG methylation change variable term (adjusted for the same terms described for the primary model) as the variance explained by that term. Finally, we conducted a pathway analysis of our top 100 findings from the genome-wide analysis using the Ingenuity Pathway analysis tool (IPA®, QIAGEN Redwood City, www.qiagen.com/ingenuity).

## Results

The characteristics of the GOLDN study population are summarized in Table 1. The present study comprises 443 participants with the mean age of 48 years. The paired *t*-test showed significant differences in participant lipid profiles following the fenofibrate intervention. On average, we observed significant decreases in TG and LDLc (*p* < 0.001) as well as a significant increase (*p* < 0.001) in HDLc.

**Table 1:** **Characteristics of the GOLDN participants (*n* = 433)**.

**Variable**	**3 weeks of fenofibrate intervention**	
	**Before**	**After**
Age (years)	48 ± 15.5	–
Sex (% female)	228 (51.5)	–
Triglycerides (mg/dl)^*^	136.42 ± 86.29	88.81 ± 49.36
HDLc (mg/dl)^*^	46.59 ± 13.03	49.69 ± 13.13
LDLc (mg/dl)^*^	123.31 ± 30.13	104.70 ± 31.57

*Data are mean ± SD and N (%)*.

* *Statistically significant difference, P < 0.001*.

**Figure 1:**
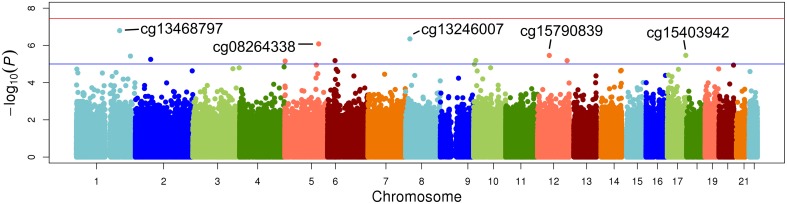
**Epigenome-wide Manhattan plot for change in DNA methylation by change in triglycerides after 3 weeks of daily fenofibrate (160 mg)**.

**Figure 2:**
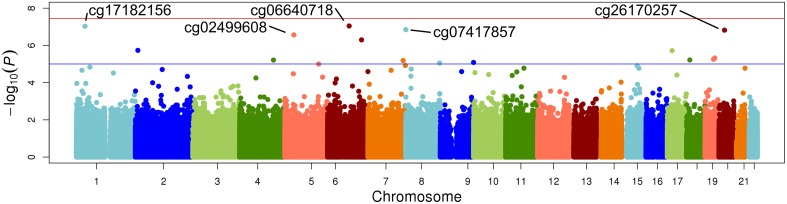
**Epigenome-wide Manhattan plot for change in DNA methylation by change in HDL after 3 weeks of daily fenofibrate (160 mg)**.

**Figure 3:**
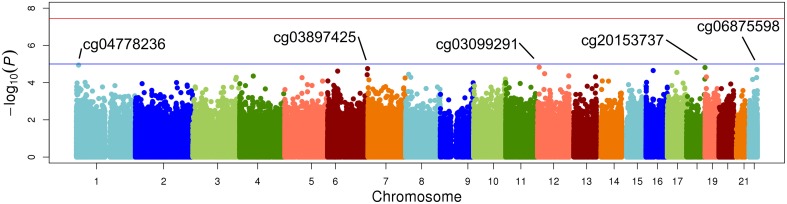
**Epigenome-wide Manhattan plot for change in DNA methylation by change in LDL after 3 weeks of daily fenofibrate (160 mg)**.

The top five results for the association between change in methylation and each individual lipid response to fenofibrate are given in Table 2. None of the associations reached the Bonferroni corrected *p*-value; however, some were close to statistical significance at that threshold after correction for multiple testing. The Manhattan plots for the genome wide analysis for each lipid response are given in Figures 1–3. The Q-Q plots for each analysis are in Supplementary Figures 1–3. Finally, results did not appreciably change upon sensitivity analysis with additional adjustment for alcohol, smoking, and dietary fat intake (see Supplemental Table 1). The variation explained by the methylation change term for results presented in Table 2 are presented in Supplemental Table 2 and range from ~3–6%. Pathway analysis of the 100 most significant CpG sites for highlighted “Triacylglycerol Biosynthesis” (*p* = 1.1^*^10^−2^) HDL-C response, “Aryl Hydrocarbon Receptor Signaling” (*p* = 3.47^*^10^−4^) for TG response, and “Sperm Motility” (*p* = 1.63^*^10^−3^) for LDL-C response.

**Table 2:** **Top five CpGs for each lipid showing change in DNA methylation for change in TG, HDLc, and LDLc profiles in the GOLDN study (*n* = 443) after 3 weeks of daily fenofibrate (160 mg)**.

**Marker**	**Chr**	**Genes**	**Location**	β (SE)^*^	***P***	**Response phenotype**
cg13468797	1	*LAMC1*	182993357	887.8 (166.7)	1.62 × 10^−7^	TG
cg13246007	8	*PCM1*	17886267	−419.5 (81.85)	4.47 × 10^−7^	TG
cg08264338	5	*NA*	143303200	−283.8 (56.76)	8.30 × 10^−7^	TG
cg15403942	17	*CBX2*	77760556	−368.7 (78.39)	3.44 × 10^−6^	TG
cg15790839	12	*MLL2*	49420118	−595.0 (126.6)	3.47 × 10^−6^	TG
cg06640718	6	*MAP3K7*	91417950	26.35 (4.847)	9.06 × 10^−8^	HDLc
cg17182156	1	*AGO1*	36393165	28.41 (5.232)	9.37 × 10^−8^	HDLc
cg07417857	8	*ERICH1*	652403	24.92 (4.659)	1.43 × 10^−7^	HDLc
cg26170257	20	*GZF1*	23345093	25.74 (4.822)	1.52 × 10^−7^	HDLc
cg02499608	5	*EGFLAM*	38402643	26.67 (5.107)	2.73 × 10^−7^	HDLc
cg04778236	1	*SPSB1*	9400739	−209.6 (47.22)	1.14 × 10^−5^	LDLc
cg03099291	12	*C12orf53*	6809939	220.3 (50.28)	1.48 × 10^−5^	LDLc
cg20153737	18	*RBFA*	77835867	−528.6 (120.9)	1.54 × 10^−5^	LDLc
cg03897425	6	*SMOC2*	169224956	−216.2 (49.81)	1.77 × 10^−5^	LDLc
cg06875598	22	*MIR4535*	49297922	−291.4 (67.52)	1.97 × 10^−5^	LDLc

Significance of P < 3.6 x 10^−8^.

* *Analysis adjusted for age, sex, and center*.

The secondary data analysis results for 104 CpGs belonging to fenofibrate pathway genes are shown in Supplementary Data Sheet 1. Methylation changes at CpGs belonging to those genes (*PPARA, APOA1, LPL, APOA5, APOC3, CETP*, and *APOB*) were also not associated with lipid response to fenofibrate in our study sample after correction for multiple testing.

## Discussion

In the current study we investigated whether changes in DNA methylation are associated with changes in lipid concentration following 3 weeks of fenofibrate treatment among 443 participants from the GOLDN study. We also considered methylation changes in known genes belonging to the pharmacodynamic pathway of fenofibrate for association lipid response in secondary analysis. Overall, our results do not support concomitant changes in DNA methylation upon a 3-week fenofibrate intervention. Nonetheless, given the strong biological plausibility of an effect of fenofibrate on DNA methylation and biological plausibility for some of our highlighted CpGs, future research is needed to expand these findings.

Despite the fact that methylation changes at top CpGs were not statistically significantly associated with lipid response to fenofibrate, a handful of markers approached genome-wide significance. Our results yielded novel genomic findings for fenofibrate response. Our most significant CpG was nearest mitogen-activated protein kinase kinase kinase 7 (*MAP3K7*) on chromosome 6 for HDL-C response (cg06640718, *p* = 9.0^*^10^−8^). The protein encoded by *MAP3K7* is a member of the serine/threonine protein kinase family and mediates signaling transduction induced by TGF beta and morphogenetic protein (BMP), and controls a variety of cell functions including transcription regulation and apoptosis. The gene has been linked to cardiovascular disease in a mouse model (Li et al., 2014). The second top finding was near argonaute RISC catalytic component 1 (*AGO1*) which plays a role in RNA interference (Hutvagner and Simard, 2008). The top finding for TG response, laminin, gamma 1 (*LAMC1*), belongs to a family of extracellular matrix glycoproteins that encode laminins. Laminins are involved in a large number of biological functions from cell adhesion, differentiation, migration, and metastasis. SplA/ryanodine receptor domain and SOCS box containing 1 (*SPSB1*), the top finding for LDL-C response, mediates the degradation of target proteins. The gene has been linked to cancer and inflammation in previous research (Liu et al., 2015). Findings related to cardiovascular disease and inflammation (*MAP3K7 and SPSB1)* could be tied to fenofibrate response, other findings are more difficult to link to the drug's mechanism (e.g., *AGO1*). Despite the lack of significance of our findings we hope to further investigate these genes as more data becomes available as part of electronic medical records linked biorepositories, other observational studies, and publically available open data repositories such as dbGaP.

There have only been a handful of drug intervention studies that have considered methylation in the response pathway. A recent 12-week intervention of aliskiren in atherosclerotic patients showed significant down regulation of miRNA in peripheral blood mononuclear cells, indicating a pathway-specific adaptation to renin inhibition (Deiuliis et al., 2014). Likewise, an 8-week intervention with flavonols among male smokers modulated the expression of genes associated with CVD. However, no major changes in DNA methylation (from leukocytes) took place (Milenkovic et al., 2014). Finally, lower methylation of two CpGs of the purinergic receptor P2Y, G-protein coupled, 12 gene (*P2Y12*) gene in leukocytes was associated with clopidogrel response in alcohol abusers suggesting that epigenetic mechanism could underlie the pathogenesis of clopidogrel resistance (Su et al., 2014). Overall, our study contributes to this growing body of literature on DNA methylation and drug response, despite the fact that we are unable to confirm whether short term changes in DNA methylation mediate response to the lipid lowering medication fenofibrate.

Our study has several limitations, which include smaller sample size and short duration of intervention where its possible that 3 weeks of fenofibrate is insufficient to observe a significant changes in DNA methylation due to drug exposure. We know from previous work in GOLDN that lipid levels associate strongly with methylation levels in the promoter regions of biologically plausible genes (Irvin et al., 2014). Therefore, we cannot exclude the possibility that changes in lipid levels with fenofibrate treatment effect changes in DNA methylation rather than the drug itself. Future studies should consider investigating the epigenetic effects of fenofibrate simultaneously for DNA methylation and histone modifications, and for a longer period of time.

## Conclusion

In the present study, no significant change in DNA methylation was found to be associated with lipid response to fenofibrate over 3 weeks. Further studies with larger sample size and longer duration of intervention will be required give further insight into possible relationships between fenofibrate treatment, lipid levels, and changes in DNA methylation.

## Author contributions

HT, DA, JO, and DKA made substantial contributions to the design of the study and the acquisition of data. MD, MI, JS, and DZ made substantial contributions to the analysis of the data. MD, MI, JS, SA, BH, DZ, HT, DA, JO, and DKA made substantial contributions to the interpretation of the data. MD drafted the manuscript and MI, JS, SA, BH, DZ, HT, DA, JO, and DKA reviewed and revised for important intellectual content. All authors reviewed and approved the submitted version of the manuscript and are accountable for the accuracy and integrity of the study.

## Funding

This study was funded by the US National Institutes of Health, National Heart, Lung and Blood Institutes grants R01 HL104135 and U01 HL72524.

### Conflict of interest statement

The authors declare that the research was conducted in the absence of any commercial or financial relationships that could be construed as a potential conflict of interest.

## Supplementary material

Supplementary Figure 1 **Q-Q Plot of change in methylation by change in Triglycerides after 3 weeks of daily fenofibrate (160 mg)**.

Supplementary Figure 2 **Q-Q Plot of change in methylation by change in HDL cholesterol after 3 weeks of daily fenofibrate (160 mg)**.

Supplementary Figure 3 **Q-Q Plot of change in methylation by change in LDL cholesterol after 3 weeks of daily fenofibrate (160 mg)**.

Supplemental Table 1 **Top five CpGs for each lipid showing change in DNA methylation for change in TG, HDLc, and LDLc profiles in the GOLDN study (*n* = 443) after 3 weeks of daily fenofibrate (160 mg) with models additionally adjusted for alcohol, smoking and fat intake**.

Supplemental Table 2 **Variability in lipid response to fenofibrate explained by the methylation change variable for results presented in Table 2 in the manuscript**.

Supplementary Data Sheet 1 **Secondary data analysis results for 104 CpGs belonging to fenofibrate pathway genes**.
