# Supplementary figures and images for: Lipid changes due to fenofibrate treatment are not associated with changes in DNA methylation patterns in the GOLDN study
Source: Front Genet. 2015 Sep 29;6:304. doi: 10.3389/fgene.2015.00304 (PMC4586504; doi:10.3389/fgene.2015.00304)

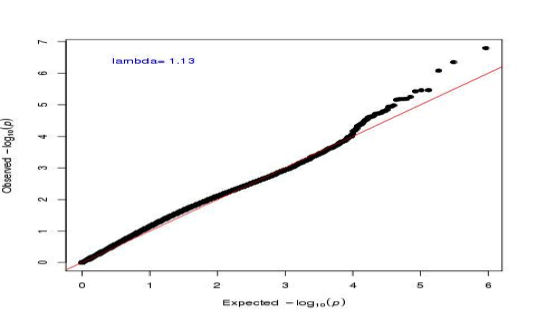

Supplement: Supplementary Figure 1 — Q-Q Plot of change in methylation by change in Triglycerides after 3 weeks of daily fenofibrate (160 mg). [file Image1.PNG]

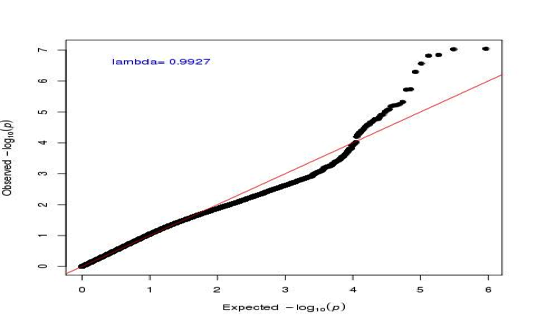

Supplement: Supplementary Figure 2 — Q-Q Plot of change in methylation by change in HDL cholesterol after 3 weeks of daily fenofibrate (160 mg). [file Image2.PNG]

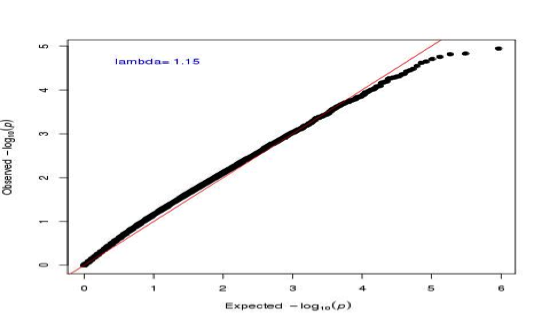

Supplement: Supplementary Figure 3 — Q-Q Plot of change in methylation by change in LDL cholesterol after 3 weeks of daily fenofibrate (160 mg). [file Image3.PNG]
