# Supplementary material for: Lipid changes due to fenofibrate treatment are not associated with changes in DNA methylation patterns in the GOLDN study
Source: Front Genet. 2015 Sep 29;6:304. doi: 10.3389/fgene.2015.00304 (PMC4586504; doi:10.3389/fgene.2015.00304)
Supplement: Supplemental Table 1 — Top five CpGs for each lipid showing change in DNA methylation for change in TG, HDLc, and LDLc profiles in the GOLDN study (n = 443) after 3 weeks of daily fenofibrate (160 mg) with models additionally adjusted for alcohol, smoking and fat intake. [file Table1.DOCX]

Supplemental Table 1: Top 5 CpGs for each lipid showing change in DNA methylation for change in TG, HDLc and LDLc profiles in the GOLDN study (n=443) after three weeks of daily fenofibrate (160 mg) with models additionally adjusted for alcohol, smoking and fat intake.

| Marker | Chr | Genes | Location | β (SE) | P | Phenotype |
| --- | --- | --- | --- | --- | --- | --- |
| cg13468797 | 1 | *LAMC1* | 182993357 | 878.7 (166.5) | 2.05x10^-7^ | Delta_TG |
| cg13246007 | 8 | *PCM1* | 17886267 | -408.6 (81.86) | 8.67x10^-7^ | Delta_TG |
| cg08264338 | 5 | *NA* | 143303200 | -277.8 (56.69) | 1.36x10^-6^ | Delta_TG |
| cg15403942 | 17 | *CBX2* | 77760556 | -357.4 (78.42) | 6.73x10^-6^ | Delta_TG |
| cg15790839 | 12 | *MLL2* | 49420118 | -588.1 (126.6) | 4.47x10^-6^ | Delta_TG |
|  |  |  |  |  |  |  |
| cg06640718 | 6 | *NA* | 91417950 | 26.35 (4.864) | 1.00x10^-7^ | Delta_HDLc |
| cg17182156 | 1 | *NA* | 36393165 | 28.49 (5.264) | 1.03×10^-7^ | Delta_HDLc |
| cg07417857 | 8 | *ERICH1* | 652403 | 24.95 (4.68) | 1.56x10^-7^ | Delta_HDLc |
| cg26170257 | 20 | *GZF1* | 23345093 | 25.69 (4.845) | 1.82x10^-7^ | Delta_HDLc |
| cg02499608 | 5 | *EGFLAM* | 38402643 | 26.79 (5.136) | 2.84x10^-7^ | Delta_HDLc |
|  |  |  |  |  |  |  |
| cg04778236 | 1 | *SPSB1* | 9400739 | -211.8 (47.35) | 9.89x10^-6^ | Delta_LDLc |
| cg03099291 | 12 | *C12orf53* | 6809939 | 221.9 (50.61) | 1.46x10^-5^ | Delta_LDLc |
| cg20153737 | 18 | NA | 77835867 | -527.6 (121.6) | 1.79x10^-5^ | Delta_LDLc |
| cg03897425 | 6 | NA | 169224956 | -218.8 (50.08) | 1.57x10^-5^ | Delta_LDLc |
| cg06875598 | 22 | NA | 49297922 | -295.1 (67.77) | 1.66x10^-5^ | Delta_LDLc |

Significance of P < 3.6x10^-8^
