# Supplementary material for: Lipid changes due to fenofibrate treatment are not associated with changes in DNA methylation patterns in the GOLDN study
Source: Front Genet. 2015 Sep 29;6:304. doi: 10.3389/fgene.2015.00304 (PMC4586504; doi:10.3389/fgene.2015.00304)
Supplement: Supplemental Table 2 — Variability in lipid response to fenofibrate explained by the methylation change variable for results presented in Table 2 in the manuscript. [file Table2.DOCX]

| CpG from Table 2. | Outcome | Variance Explained |
| --- | --- | --- |
| cg13468797 | Delta_TG | 0.0548 |
| cg13246007 | Delta_TG | 0.0543 |
| cg08264338 | Delta_TG | 0.0540 |
| cg15403942 | Delta_TG | 0.0485 |
| cg15790839 | Delta_TG | 0.0490 |
| cg06640718 | Delta_HDLc | 0.0604 |
| cg17182156 | Delta_HDLc | 0.0614 |
| cg07417857 | Delta_HDLc | 0.0581 |
| cg26170257 | Delta_HDLc | 0.0605 |
| cg02499608 | Delta_HDLc | 0.0582 |
| cg04778236 | Delta_LDLc | 0.0365 |
| cg03099291 | Delta_LDLc | 0.0383 |
| cg20153737 | Delta_LDLc | 0.0369 |
| cg03897425 | Delta_LDLc | 0.0362 |
| cg06875598 | Delta_LDLc | 0.0355 |

Supplemental Table 2. Variability in lipid response to fenofibrate explained by the methylation change variable for results presented in Table 2 in the manuscript.
